# Extended Kalman Filter-Based Vehicle Tracking Using Uniform Planar Array for Vehicle Platoon Systems

**DOI:** 10.3390/s24072351

**Published:** 2024-04-07

**Authors:** Jiho Song, Seong-Hwan Hyun

**Affiliations:** 1 Department of Electrical and Electronic Engineering, Hanyang University, ERICA, Ansan 15588, Republic of Korea; jihosong@hanyang.ac.kr; 2 School of Electrical Engineering, Seoul National University, Seoul 08826, Republic of Korea; 3 Institute of New Media & Communications (INMC), Seoul 08826, Republic of Korea

**Keywords:** vehicle-to-infrastructure, vehicle tracking, extended Kalman filter, millimeter wave, vehicle platoon systems

## Abstract

We develop an extended Kalman filter-based vehicle tracking algorithm, specifically designed for uniform planar array layouts and vehicle platoon scenarios. We first propose an antenna placement strategy to design the optimal antenna array configuration for precise vehicle tracking in vehicle-to-infrastructure networks. Furthermore, a vehicle tracking algorithm is proposed to improve the position estimation performance by specifically considering the characteristics of the state evolution model for vehicles in the platoon. The proposed algorithm enables the sharing of corrected error transition vectors among platoon vehicles, for the purpose of enhancing the tracking performance for vehicles in unfavorable positions. Lastly, we propose an array partitioning algorithm that effectively divides the entire antenna array into sub-arrays for vehicles in the platoon, aiming to maximize the average tracking performance. Numerical studies verify that the proposed tracking and array partitioning algorithms improve the position estimation performance.

## 1. Introduction

Vehicle-to-everything (V2X) is expected to connect vehicles to the internet, creating a market for connected car technology by offering innovative wireless services such as autonomous and platoon driving [1,2]. Connected car technology enables intelligent transportation services by providing access to traffic information beyond onboard sensors through wireless networks [3,4]. The evolution of fifth/sixth-generation (5G/6G) communication technologies can facilitate the high-capacity transmission and reception of data between vehicles and transmit infrastructure along roads [5]. In dynamic vehicular communication environments, the precise localization of vehicles is paramount in reliably transmitting substantial volumes of traffic information with low latency [6]. Moreover, the accurate perception of road surroundings by vehicles not only mitigates traffic congestion but also facilitates energy-efficient driving, leading to reduced power consumption [7].

Platoon driving is regarded as a promising service in connected car networks for its potential in enhancing road capacity [8,9]. Achieving ultra-reliable wireless connectivity in vehicle-to-infrastructure (V2I) networks is a crucial challenge for the evolution of vehicle platoon systems [10,11]. Although transmission and reception technologies for V2X networks have been developed based on 5G/6G, the communication environment for platoon driving scenarios exhibits distinct characteristics from typical vehicular communication networks [12,13]. To fully leverage the benefits of platoon driving, it is essential to consider diverse scenarios of platooned traffic when developing practical vehicular communication networks. Moreover, the increasing number of vehicles connected through V2I highlights the necessity of energy-efficient wireless connectivity methods utilizing limited resources. It is necessary to reevaluate vehicle tracking systems to ensure a seamless wireless connection in an energy-efficient manner and ultimately offer vehicle platoon services in V2X networks [14].

To transmit a significant volume of real-time data on road environments to vehicles, a transmitting infrastructure installed along the road must swiftly and accurately acquire the position information of the vehicles. Extensive research has been conducted on the application of Kalman filtering techniques to vehicle tracking systems. In previous studies, beacon-based vehicle tracking systems have been developed based on the extended Kalman filter (EKF) algorithm [15,16]. Additionally, the unscented Kalman filter (UKF) algorithm in [17,18,19] can be readily extended to develop vehicle tracking systems in small-sized V2I networks. To predict the trajectories of each vehicle individually, conventional tracking systems investigate the angular variations in a single spatial frequency domain using a sounding sample obtained from the ULA architecture. Although conventional tracking systems are well suited for vehicle tracking scenarios in V2I networks, several issues have not been addressed in previous studies on such systems.

In small-sized V2I networks, the distance between the roadside unit (RSU) and vehicles is significantly shorter along the *y*-axis than along the *x*-axis, leading to high sensitivity in the estimation performance regarding the *x*-axis distance [20]. Therefore, the tracking performance of RSUs significantly varies depending on the positions of the vehicles within the platoon [20]. Since the vehicles move as a group in a coordinated fashion, they share common error transitions in their transition models. Therefore, it is necessary to develop a tracking strategy that improves the average tracking performance by considering the shared transitions among vehicles. The limited wireless resources necessitate the development of a resource distribution strategy based on channel conditions for the vehicle platoon scenario [21].

Moreover, designing a cost-effective vehicle tracking system is crucial, considering the tight budget constraints of V2I networks [22]. Utilizing multiple antennas is essential in harnessing spatial beamforming gains, which is crucial in mitigating the lower expected signal-to-noise ratio (SNR) of received samples [23]. A two-dimensional (2D) uniform planar array (UPA) is considered an effective solution to accommodate multiple antennas within a limited area [24]. The vehicle tracking performance can be enhanced by exploiting angular variations in the 2D spatial frequency domains. To leverage the advantages of the 2D UPA layout, it is necessary to redesign the conventional vehicle tracking system in [16,20]. It is also imperative to develop a comprehensive analytical guideline for the design of the optimal array structure.

In this paper, we focus on developing a vehicle tracking system that is specifically tailored to the 2D antenna array layout and the state evolution model for vehicle platoon scenarios. The contributions of this paper are summarized as follows:

- We redesign a beacon signal-assisted vehicle tracking system to leverage angular variations in 2D spatial frequency domains by taking the UPA layout into consideration. Moreover, we present an analytical framework for the design of the optimal antenna array configuration from the perspective of the vehicle tracking performance. To the best of the authors’ knowledge, the analytical framework for an array configuration has not been explored with the aim of optimizing the vehicle tracking performance.
- We develop a method to fully exploit the benefits stemming from the similarities in the state transitions among the vehicles within a driving platoon. In the proposed algorithm, we focus on enhancing the tracking performance for secondary vehicles, particularly those in unfavorable positions, by leveraging the estimated information of the primary vehicle.
- We develop a strategy to effectively allocate wireless resources in a scenario whereby the vehicles in a platoon share a common frequency band for uplink channel sounding. We propose an array partitioning algorithm that adaptively subdivides the array into sub-arrays for the vehicles in the platoon by considering dynamic channel conditions.

The paper is organized as follows. In Section 2, we present the system and channel models. In Section 3, the EKF-based vehicle tracking system is proposed by considering the 2D UPA layout. In Section 4, we propose the vehicle tracking algorithm specialized for platoon driving scenarios. In Section 5, the proposed vehicle tracking systems are evaluated through numerical studies, and Section 6 details our conclusions.

*Notation:* C is the field of complex numbers, R is the field of real numbers, N is the semiring of natural numbers, and N(m,C) is the Gaussian distribution with mean vector m and covariance matrix C. Additionally, diag[·,·] is the diagonal matrix; E[·] is the expectation operator; [a,b) is the left-closed, right-open interval between *a* and *b*; ${∥\cdot ∥}_{p}$ is the *p*-norm; ∖ is the set minus operator; Re[·] is the real part of a variable; Im[·] is the imaginary part of a variable; Tr{·} is the trace of the matrix; v{·} is the principal eigenvector of the matrix; e{·} is the principal eigenvalue of the matrix; and ⊗ is the Kronecker product, 0 is the all-zeros column vector; $0_{a\times b}$ is the a×b all-zeros matrix; $I_{M}$ is the M×M identity matrix; and ${\left(a\right)}_{\ell }$ is the ℓ-th element of the column vector a. Lastly, $A^{-1}$, $A^{T}$, $A^{H}$, A(a,b), and A(:,b) denote the inverse, transpose, conjugate transpose, (a,b)-th entry, and *b*-th column of the matrix A, respectively.

## 2. System Model

**Figure 1:**
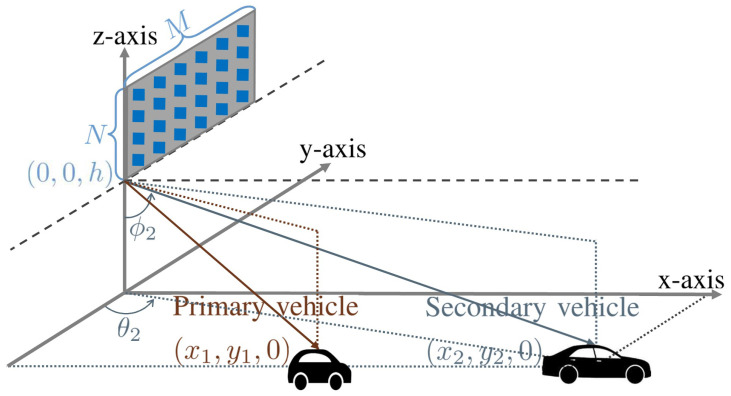
Overview of RSU communication system employing UPA.

We consider a platoon-based driving scenario in a small-sized V2I network, in which a vehicle platoon consists of multiple vehicles, u∈U={1,…,U}. From a signal-plus-angular-derivative-to-noise-ratio (SANR) perspective [20], a vehicle in a favorable position is called the primary vehicle (u=1), while vehicles in unfavorable positions are called the secondary vehicles. As depicted in Figure 1, the transmit infrastructure, i.e., the RSU, employs *M* and *N* antenna elements in the horizontal and vertical domains (T=MN) and each vehicle employs a single antenna element. A transceiver mounted on the roof of each vehicle transmits beacon signals with low power [25] for the purpose of sounding uplink channels. A substantial number of vehicles in the V2I network restricts the utilization of the available frequency spectrum primarily for vehicle tracking purposes. Therefore, we assume that the vehicles in the platoon share a common frequency band for uplink sounding to conserve frequency resources.

The received sounding sample at discrete time ℓ is combined by using zu=Re[zu]+jIm[zu]∈C1×T at the RSU. Assuming that each vehicle in the driving platoon transmits beacon signals with power $\varrho_{u}$ for uplink sounding, the combined sample for the *u*-th vehicle is defined in the complex domain

(1) $$\begin{array}{cc} r_{u}^{\ell } & =z_{u}\sum_{v\in U}\sqrt{\rho_{v}}h_{v}^{\ell }+n_{u}^{\ell }\in C, \end{array}$$

where hvℓ=Re[hvℓ]+jIm[hvℓ]∈CM is the channel vector between the RSU and the *v*-th vehicle with Re[hvℓ],Im[hvℓ]∈RM, and nuℓ=Re[nuℓ]+jIm[nuℓ] is the normalized Gaussian noise sample with Re[nuℓ],Im[nuℓ]∼N(0,12). Assuming that the path loss exponent parameter is set to two, the average SNR is defined by $\rho_{v}=\varrho_{v}{\left(\frac{\lambda }{4\pi \sigma_{n}d_{v}}\right)}^{2}$, where $\sigma_{n}^{2}$ is the power of average noise, λ is the wavelength of the radio signal, and $d_{v}$ is the distance between the RSU and the *v*-th vehicle. As outlined in [16], the position and velocity estimation process will be performed in the real domain. To utilize the acquired sample in the real domain, the sounding sample must be expressed in the real domain. Based on the domain transformation framework between real and complex domains in [26], the sounding sample in (1) is rewritten as

(2) r˜uℓ=Re[ruℓ],Im[ruℓ]T=Z˜u∑v∈Uρvh˜vℓ+n˜uℓ∈R2.

In (2), the combiner, the channel vector, and the noise vector are, respectively, defined in the real domain, such that Z˜u=Re[zu]−Im[zu]Im[zu]Re[zu]∈R2×2T, h˜vℓ=Re[hvℓ]T,Im[hvℓ]TT∈R2T, and n˜uℓ=Re[nuℓ],Im[nuℓ]T∈R2.

In a 2D UPA layout, the antenna elements are spaced by $\frac{\nu \lambda }{2}$, where ν is the element spacing parameter. Assuming that the antenna elements are arranged in a grid pattern, the spatial frequencies in the horizontal and vertical domains are defined as in [27]:

(3) $$\begin{array}{cc} \psi_{u}^{\ell } & =\nu \pi \sin\phi_{u}^{\ell }\cos\theta_{u}^{\ell }=\frac{\nu \pi y_{u}}{d_{u}}≐\psi \left(t_{u}^{\ell }\right), \end{array}$$

(4) $$\begin{array}{cc} \varphi_{u}^{\ell } & =\nu \pi \cos\phi_{u}^{\ell }=\frac{\nu \pi h}{d_{u}}≐\varphi \left(t_{u}^{\ell }\right). \end{array}$$

According to [16], the spatial frequencies are written in terms of variables in the state vector, $t_{u}^{\ell }={\left[x_{u}^{\ell },v_{u}^{\ell }\right]}^{T}$. In (3) and (4), $\theta_{u}^{\ell }$ is the horizontal angle-of-departure (AoD), $\phi_{u}^{\ell }$ is the vertical AoD, $x_{u}^{\ell }$ is the position on the *x*-axis, $y_{u}$ is the position on the *y*-axis, and $v_{u}^{\ell }$ is the velocity of the vehicle, while *h* is the height difference between the RSU and the tops of the vehicles, and $d_{u}=\sqrt{{\left(x_{u}^{\ell }\right)}^{2}+y_{u}^{2}+h^{2}}$ is the distance between the RSU and the *u*-th vehicle.

For analytical studies, we consider a single beam channel having a line-of-sight (LoS) radio path. Although we consider the simplified channel model for analysis, realistic channel vectors consisting of multiple radio paths will be used in Section 5. In the real domain, the channel vector in (2) is approximated by

(5) h˜uℓ≃h˜ψuℓ,φuℓ≐K1+KRe[βu]Re[d(ψuℓ,φuℓ)]−Im[βu]Im[d(ψuℓ,φuℓ)]Im[βu]Re[d(ψuℓ,φuℓ)]+Re[βu]Im[d(ψuℓ,φuℓ)]∈R2T,

where the small-scale fading parameter is modeled by βu=Re[βu]+jIm[βu] with Re[βu],Im[βu]∼N(0,12). The real and imaginary parts of the array response vector for the ULA layout are defined in [16]. In this paper, the procedure to define the array response vector in the real domain is extended to encompass the characteristics of the array response vectors for the UPA layout. Assuming the UPA layout, the real and imaginary parts of a ray-like beam are defined by using the real and imaginary parts of the array response vectors in both the horizontal and vertical domains, such that

Re[d(ψ,φ)]=Re[dM(ψ)]⊗Re[dN(φ)]−Im[dM(ψ)]⊗Im[dN(φ)]∈RT,Im[d(ψ,φ)]=Im[dM(ψ)]⊗Re[dN(φ)]+Re[dM(ψ)]⊗Im[dN(φ)]∈RT.

The array response vector in the real domain is defined by considering the Kronecker product three-dimensional (3D) channel model in [24,27]. For a given spatial frequency ϑ∈[−π,π), the real and imaginary parts of the array response vector having *L* antenna elements are given by

Re[dL(ϑ)]=cos(0),cos(ϑ),…,cos((L−1)ϑ)T∈RL,Im[dL(ϑ)]=sin(0),sin(ϑ),…,sin((L−1)ϑ)T∈RL.

The trajectory of the vehicles in the platoon is determined using the linear transition model outlined in [16,18]. Assuming that the sampling period is $T_{s}$, the state evolution of the *u*-th vehicle at discrete time ℓ is modeled by

(6) $$\begin{array}{c} t_{u}^{\ell }=At_{u}^{\ell -1}+o_{u}^{\ell }, \end{array}$$

where $t_{u}^{\ell }={\left[x_{u}^{\ell },v_{u}^{\ell }\right]}^{T}\in R^{2}$ is the state vector at discrete time ℓ, $A=\left[\begin{array}{cc} 1 & T_{s}\\ 0 & 1 \end{array}\right]\in R^{2\times 2}$ is the state transition matrix, and $o_{u}^{\ell }\in R^{2}$ is the error transition vector of the *u*-th vehicle, which the RSU cannot predict. We now model the unpredictable movements of the vehicles in the platoon based on a Gaussian distribution [28,29]. The unpredictable movement that is common for all vehicles in the platoon is modeled by $b^{\ell }∼N\left(0,Q_{b}\right)$. Although the vehicles in the platoon move as a group in a coordinated fashion, sudden changes in road environments can lead to the unpredictable movement of individual vehicles. The unpredictable movement that is specialized for the secondary vehicle is modeled by $c^{\ell }∼N\left(0,Q_{c}\right)$. By considering the unpredictable movements of vehicles, the error transition vectors of the primary and secondary vehicles are modeled as $o_{1}^{\ell }=b^{\ell }$ and $o_{2}^{\ell }=b^{\ell }+c^{\ell }$, respectively. In accordance with [16,20], the covariance matrices are modeled by the following.

$$\begin{array}{c} Q_{b}=\sigma_{b}^{2}\mathrm{diag}\left[T_{s}^{2},1\right]\;\;\;\;\;\mathrm{and}\;\;\;\;\;Q_{c}=\sigma_{c}^{2}\mathrm{diag}\left[T_{s}^{2},1\right]. \end{array}$$

Notice that $Q_{o_{1}}=Q_{b}$ and $Q_{o_{2}}=Q_{b}+Q_{c}$. We assume that the error variances follow $\sigma_{b}^{2}\gg \sigma_{c}^{2}$.

In this paper, we utilize the classical Kalman filtering algorithm to address the Gaussian error model. Future research could explore practical vehicle tracking systems capable of handling non-Gaussian error models using non-Gaussian Kalman filtering techniques as in [30,31]. Additionally, it would be an intriguing future research topic to develop vehicle tracking systems based on the extended H-infinity filter, considering scenarios where the distributions of the state transition and observation errors are not known at the RSU, and scenarios involving cyber-attacks that interfere with the accurate state estimation of vehicles [32,33,34].

## 3. EKF-Based Vehicle Tracking System Using 2D UPA Layout

Our goal is to enhance the tracking performance of beacon-based vehicle tracking systems. First, we aim to improve the effective SNR of the received sounding samples in (2). A transceiver installed on a vehicle is designed using cost-effective, low-power components by considering tight budget constraints. The lower expected SNR of the received samples at the RSU necessitates the utilization of a larger-sized antenna array. The UPA is considered to be a solution for the efficient deployment of a large number of antenna elements in a limited area. Second, we seek to precisely monitor the variations in the spatial frequency domains. In the beacon-based vehicle tracking framework [16,20], these variations in the spatial frequency domains are employed to track vehicle movements on the road. The 2D structure of the UPA layout can be utilized to fully exploit the angular variations by monitoring both the horizontal and vertical spatial frequency domains.

The objective of this section is to develop a beacon-based vehicle tracking system that can enhance the vehicle tracking performance by exploiting the full benefits of the 2D antenna array layout. In Section 3.1, we first redesign the EKF-based vehicle tracking algorithm in [16,20] by taking the UPA layout into consideration. In Section 3.2, we next establish an analytical framework to optimize the UPA layout to maximize the vehicle tracking performance.

Before presenting the proposed algorithms, we provide a summary of the symbols used to denote the key variables.

- The symbol $\tilde{v}$ is used to denote the variable in the real domain, transformed from the variable v in the complex domain.
- The symbol ${\dot{v}}_{\upsilon }$ is used to denote the derivative of $\tilde{v}$ with respect to υ.
- The symbol ${\hat{\cdot }}^{\ell |\ell -1}$ is used to denote the predicted variables following the ℓ-th state prediction process.
- The symbol ${\hat{\cdot }}^{\ell }$ is used to denote the estimated variables following the ℓ-th state update process.
- The symbols $\overset{˘}{\cdot }$ and $\overset{ˇ}{\cdot }$ are used to denote, respectively, the dummy variables in an optimization problem and the solution to the problem.

### 3.1. Proposed UPA-Based Vehicle Tracking System Sharing Common Frequency

In this section, we review the previously reported vehicle tracking systems in [16]. Before initiating the Kalman filtering process, an initial state vector, $t_{u}^{0}={\left[x_{u}^{0},v_{u}^{0}\right]}^{T}$, is transmitted to the RSU through the feedback link. To address the initial feedback error stemming from the time delay of the feedback process, we include a Gaussian error term in the initial state vector. The Gaussian error term is modeled as $e_{u}^{\epsilon }=\epsilon t_{u}^{0}$, where $\epsilon ∼N(0,\sigma_{\epsilon }^{2}T_{s}^{2})$. The RSU then begins the EKF-based vehicle tracking process by using the contaminated initial state vector, ${\hat{t}}_{u}^{0}=t_{u}^{0}+e_{u}^{\epsilon }$.

In the state prediction process, the state vector and the covariance are predicted based on the Kalman filter-based tracking framework in [35], such that

$$\begin{array}{c} {\hat{t}}_{u}^{\ell |\ell -1}=A{\hat{t}}_{u}^{\ell -1}\;\;\;\;\;\mathrm{and}\;\;\;\;\;{\hat{Q}}_{u}^{\ell |\ell -1}=A{\hat{Q}}_{u}^{\ell -1}A^{T}+Q_{o_{u}}, \end{array}$$

where $Q_{o_{u}}$ denotes the covariance of the error transition vector. The channel is then predicted based on the approximated channel vector formulation in (5), such that

(7) $$\begin{array}{c} {\hat{h}}_{u}^{\ell |\ell -1}=\tilde{h}\left(\psi ,\varphi \right){|}_{\left(\psi ,\varphi \right)=\left(\psi \left({\hat{t}}_{u}^{\ell |\ell -1}\right),\varphi \left({\hat{t}}_{u}^{\ell |\ell -1}\right)\right)}\in R^{2T}. \end{array}$$

In (7), the spatial frequencies are as defined in (3) and (4) by using position variables in the predicted state vector, ${\hat{t}}_{u}^{\ell |\ell -1}$.

Based on the Kalman filtering framework in [35,36], in the state update process, the predicted state vector and the covariance matrix are updated by using the received sample, ${\tilde{r}}_{u}$, in (2), such that

(8) $$\begin{array}{cc} {\hat{t}}_{u}^{\ell } & ={\hat{t}}_{u}^{\ell |\ell -1}+{\tilde{K}}_{u}\left({\tilde{r}}_{u}-{\tilde{Z}}_{u}\sum_{v\in U}\sqrt{\rho_{v}}{\hat{h}}_{v}^{\ell |\ell -1}\right), \end{array}$$

(9) $$\begin{array}{cc} {\hat{Q}}_{u}^{\ell } & =\left(I_{2}-\sqrt{\rho_{u}}{\tilde{K}}_{u}{\tilde{Z}}_{u}{\tilde{D}}_{u}\right){\hat{Q}}_{u}^{\ell |\ell -1}, \end{array}$$

where ${\tilde{K}}_{u}$ is the Kalman gain matrix. However, the channel sounding model in (2) is not a linear system because the channel vector, ${\hat{t}}_{u}^{\ell }$, cannot be defined linearly in terms of position variables in the state vector. In the EKF framework, the single beam channel is thus linearly approximated by ${\tilde{h}}_{u}^{\ell }≃{\hat{h}}_{u}^{\ell |\ell -1}+{\tilde{D}}_{u}e_{u}^{\ell |\ell -1}$, where $e_{u}^{\ell |\ell -1}=t_{u}^{\ell }-{\hat{t}}_{u}^{\ell |\ell -1}$ represents the error of the predicted state vector.

Next, to expand the existing system to incorporate the use of the UPA layout, we compute the Jacobian matrix of the approximated channel, D˜u=Re[Du]T,Im[Du]TT, by considering both the horizontal and vertical spatial frequency domains. The Jacobian matrix of the approximated channel for the ULA is defined in [16]. In this paper, the procedure to define the Jacobian matrix is extended to encompass the characteristics of the approximated channel for the UPA layout. For a given predicted state vector ${\hat{t}}_{u}^{\ell |\ell -1}$, the Jacobian matrix is defined by using the derivatives of the channel vector that represents the angular variations in both the horizontal and vertical spatial frequency domains, such that

(10) $$\begin{array}{cc} {\tilde{D}}_{u} & ={\dot{h}}_{\psi }\left(\psi ,\varphi \right){|}_{\left(\psi ,\varphi \right)=\left(\psi \left({\hat{t}}_{u}^{\ell |\ell -1}\right),\varphi \left({\hat{t}}_{u}^{\ell |\ell -1}\right)\right)}{\dot{\psi }}_{t}{\left(t\right)}^{T}{|}_{t={\hat{t}}_{u}^{\ell |\ell -1}}\\ \; & \;+\;{\dot{h}}_{\varphi }\left(\psi ,\varphi \right){|}_{\left(\psi ,\varphi \right)=\left(\psi \left({\hat{t}}_{u}^{\ell |\ell -1}\right),\varphi \left({\hat{t}}_{u}^{\ell |\ell -1}\right)\right)}{\dot{\varphi }}_{t}{\left(t\right)}^{T}{|}_{t={\hat{t}}_{u}^{\ell |\ell -1}}. \end{array}$$

In (10), the derivatives of the spatial frequencies over the state vectors are, respectively, defined by

$$\begin{array}{c} {\dot{\psi }}_{t}\left(t\right)=\frac{-\nu \pi xy}{{\left(x^{2}+y^{2}+h^{2}\right)}^{\frac{3}{2}}}\left[\begin{array}{c} 1\\ T_{s} \end{array}\right]\;\;\;\;\;\mathrm{and}\;\;\;\;\;{\dot{\varphi }}_{t}\left(t\right)=\frac{-\nu \pi xh}{{\left(x^{2}+y^{2}+h^{2}\right)}^{\frac{3}{2}}}\left[\begin{array}{c} 1\\ T_{s} \end{array}\right]. \end{array}$$

Furthermore, the derivatives of the channel vector over the spatial frequencies are defined by

h˙ψ(ψ,φ)=K1+KRe[β]Re[d˙ψ(ψ,φ)]−Im[β]Im[d˙ψ(ψ,φ)]Re[β]Im[d˙ψ(ψ,φ)]+Im[β]Re[d˙ψ(ψ,φ)]∈R2T,h˙φ(ψ,φ)=K1+KRe[β]Re[d˙φ(ψ,φ)]−Im[β]Im[d˙φ(ψ,φ)]Re[β]Im[d˙φ(ψ,φ)]+Im[β]Re[d˙φ(ψ,φ)]∈R2T.

The derivatives of the real and imaginary parts of the ray-like beam over ψ are defined by

(11) Re[d˙ψ(ψ,φ)]=Re[d˙ψM(ψ)]⊗Re[dN(φ)]−Im[d˙ψM(ψ)]⊗Im[dN(φ)]∈RT,

(12) Im[d˙ψ(ψ,φ)]=Im[d˙ψM(ψ)]⊗Re[dN(φ)]+Re[d˙ψM(ψ)]⊗Im[dN(φ)]∈RT,

where the real and imaginary parts of the derivatives of the array response vectors are given by

Re[d˙ϑL(ϑ)]=−0,sin(ϑ),…,(L−1)sin((L−1)ϑ)T∈RL,Im[d˙ϑL(ϑ)]=0,cos(ϑ),…,(L−1)cos((L−1)ϑ)T∈RL.

The derivatives of the real and imaginary parts of the ray-like beam over φ can be defined by using a similar method as in (11) and (12).

Lastly, we design the Kalman gain matrix and the combiner in (8) and (9) to correct the unpredictable movement of a vehicle while suppressing the interference signals. Similar to [36,37], the Kalman gain matrix is designed to minimize the trace of the error covariance matrix, ${\hat{Q}}_{u}^{\ell }=E\left[e_{u}^{\ell }{\left(e_{u}^{\ell }\right)}^{T}\right]$ with $e_{u}^{\ell }=t_{u}^{\ell }-{\hat{t}}_{u}^{\ell }$, such that

$$\begin{array}{c} {\tilde{K}}_{u}=\sqrt{\rho_{u}}{\hat{Q}}_{u}^{\ell |\ell -1}{\tilde{D}}_{u}^{T}{\tilde{Z}}_{u}^{T}{\left({\tilde{Z}}_{u}\left(\sum_{v\in U}\rho_{v}{\tilde{D}}_{v}{\hat{Q}}_{v}^{\ell |\ell -1}{\tilde{D}}_{v}^{T}\right){\tilde{Z}}_{u}^{T}+\left(\frac{\sum_{v\in U}\rho_{v}}{K+1}+1\right)\frac{I_{2}}{2}\right)}^{-1}. \end{array}$$

The combiner at the RSU is designed to maximize the power of the desired sounding signal for each vehicle, while suppressing the interference signals from other vehicles and the signals through the non-line-of-sight (NLoS) radio paths. In this paper, we aim to compute a combiner that can minimize the updated covariance matrix, ${\hat{Q}}_{u}^{\ell }$, in (9). The minimization problem of ${\hat{Q}}_{u}^{\ell }$ can be reformulated as the maximization problem ${arg min}_{{\overset{˘}{Z}}_{u}}\mathrm{Tr}\left\{\left(I_{2}-\sqrt{\rho_{u}}{\tilde{K}}_{u}{\overset{˘}{Z}}_{u}{\tilde{D}}_{u}\right){\hat{Q}}_{u}^{\ell |\ell -1}\right\}={arg max}_{{\overset{˘}{Z}}_{u}}\mathrm{Tr}\left\{\sqrt{\rho_{u}}{\tilde{K}}_{u}{\overset{˘}{Z}}_{u}{\tilde{D}}_{u}{\hat{Q}}_{u}^{\ell |\ell -1}\right\}$, as in [16]. Therefore, the combiner is computed to maximize the trace of the following matrix, which can be defined by plugging the Kalman gain matrix, such as

(13) $$\begin{array}{cc} \; & \mathrm{Tr}\left\{\rho_{u}{\tilde{\Lambda }}^{-1}{\tilde{Z}}_{u}{\tilde{D}}_{u}\left(:,1\right){\left({\hat{Q}}_{u}^{\ell |\ell -1}\left(1,1\right)\right)}^{2}{\tilde{D}}_{u}{\left(:,1\right)}^{T}{\tilde{Z}}_{u}^{T}\right\},\;\;\;\;\;\mathrm{with}\\ \; & \tilde{\Lambda }={\tilde{Z}}_{u}\underset{\left(a\right)}{\left(\underset{︸}{\sum_{v\in U}\rho_{v}{\tilde{D}}_{v}\left(:,1\right){\hat{Q}}_{v}^{\ell |\ell -1}\left(1,1\right){\tilde{D}}_{v}{\left(:,1\right)}^{T}}\right)}{\tilde{Z}}_{u}^{T}+\left(\underset{\left(b\right)}{\underset{︸}{\frac{\sum_{v\in U}\rho_{v}}{K+1}}}+1\right)\frac{I_{2}}{2}, \end{array}$$

where $\sum_{v\in U\setminus \{u\}}\rho_{v}{\tilde{D}}_{v}\left(:,1\right){\hat{Q}}_{v}^{\ell |\ell -1}\left(1,1\right){\tilde{D}}_{v}{\left(:,1\right)}^{T}$ in (a) denotes the power of the inter-user interference terms that must be suppressed by using a combiner at the RSU, and $\frac{\sum_{v\in U}\rho_{v}}{K+1}$ in (b) denotes the power of the interference signal that is transmitted through NLoS radio paths. In this paper, we select the (1,1)-th component of the covariance matrix and the first column vector of the Jacobian matrix to focus on minimizing the position error. It is difficult to compute the optimal combiner in the real domain so we reformulate the optimization problem in the complex domain [16]. Based on the Rayleigh quotient method in [38], the combiner is computed by solving the reformulated maximization problem defined in the complex domain,

(14) zu=Re[zu]+jIm[zu]=arg maxz˘∈C1×Tz˘(ρuDu(:,1)(Q^uℓ|ℓ−1(1,1))2Du(:,1)H)z˘Hz˘Λz˘H=vρuΛ−1Du(:,1)(Q^uℓ|ℓ−1(1,1))2Du(:,1)H∈C1×T,

where $\Lambda =\sum_{v\in U}\rho_{v}D_{v}\left(:,1\right){\hat{Q}}_{v}^{\ell |\ell -1}\left(1,1\right)D_{v}{\left(:,1\right)}^{H}+\left(\frac{\sum_{v\in U}\rho_{v}}{K+1}+1\right)I_{T}$, and Du=Re[Du]+jIm[Du]. The combiner in the real domain is then computed as Z˜u=Re[zu]−Im[zu]Im[zu]Re[zu]∈R2×2T. We assume that the RSU computes the combining vector in (1) for every $\Omega T_{s}$ seconds, where Ω represents the combiner switching parameter. In this paper, we adopt the partially connected hybrid beamforming systems from [39,40]. Throughout this paper, all combining vectors will be redefined to adhere to the power constraints of hybrid beamforming systems.

### 3.2. Optimal Configuration of Antenna Array for Vehicle Tracking

The antenna elements comprising the antenna array can be arranged in various configurations. It is required to develop an analytical framework that can evaluate antenna array configurations from the perspective of the vehicle tracking performance. Assuming that the total number of antennas is T=MN and the area of the antenna array is limited, we aim to design the optimal antenna array structure that maximizes the vehicle tracking performance. In [20], the SANR metric is derived to assess vehicle tracking systems that estimate vehicle positions by leveraging the angular variations resulting from vehicle movements. It is confirmed that the SANR metric is effective in evaluating the variations in the spatial frequency domains caused by vehicle movement. In this paper, the procedure to define the SANR metric for the ULA is extended to monitor the angular variations in both the horizontal and vertical domains, assuming the UPA layout. In the following proposition, we derive the SANR metric to evaluate configurations of 2D antenna arrays, by taking the UPA layout into consideration.

**Proposition 1.**  *Assuming that an RSU employs M and N antenna elements in the horizontal and vertical domains, the SANR of the sounding sample is defined by*

$$\begin{array}{c} \gamma \left(M,N\right)=\kappa \left(\left(M-1\right)\left(2M-1\right)y^{2}+\left(N-1\right)\left(2N-1\right)h^{2}\right), \end{array}$$

*where $\kappa =\frac{\varrho K\nu^{2}\lambda^{2}x^{2}\hat{Q}\left(1,1\right)}{6\left(K+1\right){\left(4\sigma_{n}\right)}^{2}{\left(x^{2}+y^{2}+h^{2}\right)}^{4}}$.*

**Proof** **of** **Proposition 1.** We take a closer look at the difference between the sounding sample and the predicted sample in (8) to quantify the angular variations owing to vehicle movements. Similar to [20], we derive the SANR of the UPA-installed vehicle tracking system by computing the expectation of the norm square of the state-error-correction component, $\sqrt{\rho }\tilde{Z}\tilde{D}e$ (the index of the vehicle is dropped to simplify the presentation), such that

(15) $$\begin{array}{cc} \gamma & =\rho E\left[∥\tilde{Z}\tilde{D}{e∥}_{2}^{2}\right]=E\left[e^{T}{\tilde{D}}^{T}{\tilde{Z}}^{T}\tilde{Z}\tilde{D}e\right]\\ \; & =\frac{\rho }{NM}E\left[e^{T}\left({\dot{\psi }}_{t}{\dot{h}}_{\psi }^{T}+{\dot{\varphi }}_{t}{\dot{h}}_{\varphi }^{T}\right)\left({\dot{h}}_{\psi }{\dot{\psi }}_{t}^{T}+{\dot{h}}_{\varphi }{\dot{\varphi }}_{t}^{T}\right)e\right]\\ \; & \overset{\left(a\right)}{=}\frac{\rho }{NM}\left(E\left[e^{T}{\dot{\psi }}_{t}{\dot{h}}_{\psi }^{T}{\dot{h}}_{\psi }{\dot{\psi }}_{t}^{T}e\right]+E\left[e^{T}{\dot{\varphi }}_{t}{\dot{h}}_{\varphi }^{T}{\dot{h}}_{\varphi }{\dot{\varphi }}_{t}^{T}e\right]\right)\\ \; & \overset{\left(b\right)}{=}\frac{\rho K}{6(K+1)}\left(\left(M-1\right)\left(2M-1\right)E\left[e^{T}{\dot{\psi }}_{t}{\dot{\psi }}_{t}^{T}e\right]+\left(N-1\right)\left(2N-1\right)E\left[e^{T}{\dot{\varphi }}_{t}{\dot{\varphi }}_{t}^{T}e\right]\right)\\ \; & \overset{\left(c\right)}{≃}\frac{\rho K\nu^{2}x^{2}\pi^{2}\hat{Q}\left(1,1\right)}{6\left(K+1\right){\left(x^{2}+y^{2}+h^{2}\right)}^{3}}\left(\left(M-1\right)\left(2M-1\right)y^{2}+\left(N-1\right)\left(2N-1\right)h^{2}\right)\\ \; & \overset{\left(d\right)}{=}\frac{\varrho K\nu^{2}x^{2}\hat{Q}\left(1,1\right)}{6\left(K+1\right){\left(4\sigma_{n}\right)}^{2}{\left(x^{2}+y^{2}+h^{2}\right)}^{4}}\left(\left(M-1\right)\left(2M-1\right)y^{2}+\left(N-1\right)\left(2N-1\right)h^{2}\right). \end{array}$$

In (15), (a) is derived with $E\left[{\dot{h}}_{\psi }^{T}{\dot{h}}_{\varphi }\right]=0$, (b) is derived with

Eh˙ψTh˙ψ=E|Re[β]|2+|Im[β]|2∥Re[d˙ψ(ψ,φ)]∥22+∥Im[d˙ψ(ψ,φ)]∥22=∥Re[d˙ψM(ψ)]⊗Re[d˜N(φ)]∥22+∥Im[d˙ψM(ψ)]⊗Im[d˜N(φ)]∥22+∥Im[d˙ψM(ψ)]⊗Re[d˜N(φ)]∥22+∥Re[d˙ψM(ψ)]⊗Im[d˜N(φ)]∥22=∑ℓ=0N−1cos2ℓφ+sin2ℓφRe[∥d˙ψM(ψ)]∥22+∥Im[d˙ψM(ψ)]∥22=∑ℓ=0N−1cos2ℓφ+sin2ℓφ∑m=0M−1m2(sin2mψ+cos2mψ)=NM(M−1)(2M−1)6,

and $E\left[{\dot{h}}_{\varphi }^{T}{\dot{h}}_{\varphi }\right]=\frac{MN(N-1)(2N-1)}{6}$ with $\sum_{\ell =0}^{n}\ell^{2}=\frac{n(n+1)(2n+1)}{6}$. In addition, (c) is derived with

$$\begin{array}{c} {\dot{\psi }}_{t}{\dot{\psi }}_{t}^{T}≃\left[\begin{array}{cc} \frac{\nu^{2}\pi^{2}x^{2}y^{2}}{{\left(x^{2}+y^{2}+h^{2}\right)}^{3}} & 0\\ 0 & 0 \end{array}\right]\;\;\;\;\;\mathrm{and}\;\;\;\;\;{\dot{\varphi }}_{t}{\dot{\varphi }}_{t}^{T}≃\left[\begin{array}{cc} \frac{\nu^{2}\pi^{2}x^{2}h^{2}}{{\left(x^{2}+y^{2}+h^{2}\right)}^{3}} & 0\\ 0 & 0 \end{array}\right], \end{array}$$

which are approximated by assuming $T_{s}\ll 1$ [20]. Lastly, (d) is derived because the average SNR is given by $\rho =\varrho {\left(\frac{\lambda }{4\sigma_{n}d}\right)}^{2}$ with $d=\sqrt{x^{2}+y^{2}+h^{2}}$. Finally, the SANR in (15) is rewritten as

$$\begin{array}{c} \gamma \left(M,N\right)=\kappa \left(\left(M-1\right)\left(2M-1\right)y^{2}+\left(N-1\right)\left(2N-1\right)h^{2}\right), \end{array}$$

where $\kappa =\frac{\varrho K\nu^{2}\lambda^{2}x^{2}\hat{Q}\left(1,1\right)}{6\left(K+1\right){\left(4\sigma_{n}\right)}^{2}{\left(x^{2}+y^{2}+h^{2}\right)}^{4}}$. □

The vehicle tracking performance of the system in Section 3.2 is proportional to the value of the SANR metric in Proposition 1. The optimal configuration of the antenna array can be determined by maximizing the SANR, such that

(16) $$\begin{array}{cc} \overset{ˇ}{M} & =\underset{\overset{˘}{M}\in M}{arg max}\gamma \left(\overset{˘}{M},\frac{T}{\overset{˘}{M}}\right)\\ \; & =\underset{\overset{˘}{M}\in M}{arg max}\left(2{\overset{˘}{M}}^{2}-3\overset{˘}{M}+1\right){\bar{y}}^{2}+\left(\frac{2T^{2}}{{\overset{˘}{M}}^{2}}-\frac{3T}{\overset{˘}{M}}+1\right)h^{2}, \end{array}$$

where the object function is written by plugging $\overset{˘}{N}=\frac{T}{\overset{˘}{M}}$ into the SANR. In (16), M denotes the set of possible divisors of the number of antenna elements, *T*, and the squared position variable in the *y*-axis is replaced by its arithmetic mean, ${\bar{y}}^{2}=E\left[y^{2}\right]$. Notice that the common term κ is dropped for simplicity.

We now compute the second derivative of an object function to check the convexity of the function. The second derivative of the object function is computed as

$$\begin{array}{cc} \frac{d^{2}\left(\left(2M^{2}-3M+1\right){\bar{y}}^{2}+\left(\frac{2T^{2}}{M^{2}}-\frac{3T}{M}+1\right)h^{2}\right)}{dM^{2}} & =\frac{d\left(\left(4M-3\right){\bar{y}}^{2}-\left(\frac{4T^{2}}{M^{3}}-\frac{3T}{M^{2}}\right)h^{2}\right)}{dM}\\ \; & =\frac{6Th^{2}\left(2T-M\right)}{M^{4}}+4M{\bar{y}}^{2}\overset{\left(a\right)}{>}0, \end{array}$$

where (a) is derived because 2T>M>0. The object function is convex because its second derivative is nonnegative. Therefore, the maximum value of the SANR will be obtained when $\overset{ˇ}{M}=1$ or $\overset{ˇ}{M}=T$.

First, we determine the optimum configuration of a one-dimensional (1D) ULA layout, such that

(17) $$\begin{array}{c} \left(\overset{ˇ}{M},\overset{ˇ}{N}\right)=\{\begin{array}{cc} (T,1), & {\bar{y}}^{2}\geq h^{2}\\ (1,T), & {\bar{y}}^{2}<h^{2} \end{array}, \end{array}$$

because the feasible set of the optimization problem in (16) is rewritten as M∈{1,T}. Second, the optimal configuration of the 2D UPA layout is determined as

(18) $$\begin{array}{c} \begin{array}{cc} \mathrm{Horizontal}\mathrm{UPA}\;\;\;(\overset{ˇ}{M}>\overset{ˇ}{N}>1), & {\bar{y}}^{2}\geq h^{2}\\ \mathrm{Vertical}\mathrm{UPA}\;\;\;(\overset{ˇ}{N}>\overset{ˇ}{M}>1), & {\bar{y}}^{2}<h^{2} \end{array}, \end{array}$$

because the feasible set of the optimization problem in (16) is rewritten as $\overset{˘}{M}\in M\setminus \left\{1,T\right\}$.

If there is ample space for antenna element deployment, it would be more advantageous to use a 1D ULA for vehicle tracking compared to a 2D UPA. Furthermore, it is verified that the horizontal ULA ($\overset{ˇ}{M}=T$) is better than the vertical ULA ($\overset{ˇ}{N}=T$) in enhancing the vehicle tracking performance because ${\bar{y}}^{2}>h^{2}$ in most realistic road environments. If there were no restrictions on the space available for antenna array installation, it would be better to utilize the ULA for vehicle tracking. However, the limited area of the antenna necessitates the use of the 2D UPA, which can host many antenna elements in a grid pattern.

**Figure 2:**
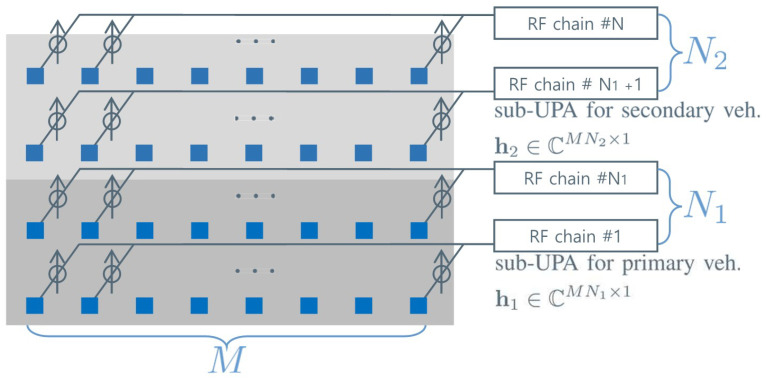
Partially connected hybrid beamforming architecture using UPA.

In this paper, we consider the rectangular horizontal UPA with a longer length horizontally than vertically, i.e., M>N>1. To provide a detailed description of the UPA designed based on the proposed array configuration framework, Figure 2 gives a close-up view of the antenna array at the RSU, extracted from Figure 1, where the small square represents the antenna elements and the circled arrow indicates the radio frequency (RF) phase shifters.

Furthermore, we consider the partially connected hybrid beamforming architecture using the UPA with *N* RF chains, in which *M* antenna elements in each row of the horizontal domain are connected to a single RF chain [40]. It should be noted that the hybrid beamforming architecture is regarded as the beamforming and sensing solution for the development of integrated communication and sensing systems [39]. As depicted in Figure 2, each row of the horizontal domain contains an RF chain, enabling individual analog beamforming/combining through a set of phase shifters.

## 4. Vehicle Tracking Algorithm for Platoon Systems

In small-sized V2I networks, the distance between the RSU and vehicles is significantly shorter on the *y*-axis compared to the *x*-axis. This disparity leads to the heightened sensitivity of the vehicle estimation performance to the distance on the *x*-axis. The vehicle tracking system in [16] demonstrates excellent estimation performance for the primary vehicle in tracking-friendly positions, but its accuracy notably declines when applied to secondary vehicles in unfavorable positions.

Before discussing the vehicle tracking performance, we discuss the state transition model specifically designed for vehicle platoon scenarios. The platoon vehicles are synchronized with each other, resulting in similarities in their driving state transitions. Therefore, the state transition vector of the secondary vehicle can be written in terms of the state vector of the primary vehicle and the state difference vector, such that

(19) $$\begin{array}{cc} t_{2}^{\ell } & =t_{1}^{\ell }+t_{d}^{\ell }=A\left(t_{1}^{\ell -1}+t_{d}^{\ell -1}\right)+\left(b^{\ell }+c^{\ell }\right)\\ \; & =\left(At_{1}^{\ell -1}+b^{\ell }\right)+\left(At_{d}^{\ell -1}+c^{\ell }\right), \end{array}$$

where the error transition vectors of the primary and secondary vehicles are, respectively, modeled by $o_{1}^{\ell }=b^{\ell }$ and $o_{2}^{\ell }=b^{\ell }+c^{\ell }$ with $b^{\ell }∼N\left(0,Q_{b}\right)$ and $c^{\ell }∼N\left(0,Q_{c}\right)$. The covariance matrix of the secondary vehicle can then be rewritten as

$$\begin{array}{cc} {\hat{Q}}_{2}^{\ell } & =E\left[\left(t_{2}^{\ell }-{\hat{t}}_{2}^{\ell }\right){\left(t_{2}^{\ell }-{\hat{t}}_{2}^{\ell }\right)}^{T}\right]=E\left[\left(\left(t_{1}^{\ell }-{\hat{t}}_{1}^{\ell }\right)+\left(t_{d}^{\ell }-{\hat{t}}_{d}^{\ell }\right)\right){\left(\left(t_{1}^{\ell }-{\hat{t}}_{1}^{\ell }\right)+\left(t_{d}^{\ell }-{\hat{t}}_{d}^{\ell }\right)\right)}^{T}\right]\\ \; & \overset{\left(a\right)}{=}E\left[\left(t_{1}^{\ell }-{\hat{t}}_{1}^{\ell }\right){\left(t_{1}^{\ell }-{\hat{t}}_{1}^{\ell }\right)}^{T}\right]+E\left[\left(t_{d}^{\ell }-{\hat{t}}_{d}^{\ell }\right){\left(t_{d}^{\ell }-{\hat{t}}_{d}^{\ell }\right)}^{T}\right]={\hat{Q}}_{1}^{\ell }+{\hat{Q}}_{d}^{\ell }, \end{array}$$

where (a) is derived because $t_{1}^{\ell }$ and $t_{d}^{\ell }$ are statistically independent.

The vehicle tracking performance, after conducting the ℓ-th state update process, can be evaluated by using the traces of the updated error covariance matrices. Based on the Kalman filtering framework in [35,36], the covariance matrices are updated as

(20) $$\begin{array}{cc} {\hat{Q}}_{1}^{\ell } & =\left(I_{2}-\sqrt{\rho_{1}}{\tilde{K}}_{1}{\tilde{Z}}_{1}{\tilde{D}}_{1}\right)(\underset{{\hat{Q}}_{1}^{\ell |\ell -1}}{\underset{︸}{A{\hat{Q}}_{1}^{\ell -1}A^{T}+Q_{b}}}), \end{array}$$

(21) $$\begin{array}{cc} {\hat{Q}}_{2}^{\ell } & \overset{\left(a\right)}{=}\left(I_{2}-\sqrt{\rho_{2}}{\tilde{K}}_{2}{\tilde{Z}}_{2}{\tilde{D}}_{2}\right)(\underset{{\hat{Q}}_{1}^{\ell |\ell -1}}{\underset{︸}{A{\hat{Q}}_{1}^{\ell -1}A^{T}+Q_{b}}}+\underset{{\hat{Q}}_{d}^{\ell |\ell -1}}{\underset{︸}{A{\hat{Q}}_{d}^{\ell -1}A^{T}+Q_{c}}}), \end{array}$$

where (a) is derived because the state difference vector and the covariance matrix are predicted as

(22) $$\begin{array}{c} {\hat{t}}_{d}^{\ell |\ell -1}=A{\hat{t}}_{d}^{\ell -1}\;\;\;\;\;\mathrm{and}\;\;\;\;\;{\hat{Q}}_{d}^{\ell |\ell -1}=A{\hat{Q}}_{d}^{\ell -1}A^{T}+Q_{c}. \end{array}$$

In the state update process of the conventional system in Section 3.1, the error terms in ${\hat{Q}}_{1}^{\ell |\ell -1}$ of the primary vehicle will be corrected by using the high SANR sounding sample, while the error terms in ${\hat{Q}}_{2}^{\ell |\ell -1}={\hat{Q}}_{1}^{\ell |\ell -1}+{\hat{Q}}_{d}^{\ell |\ell -1}$ of the secondary vehicle will be corrected by using the low SANR sounding sample.

The values of the updated error covariances at discrete time ℓ depend on the SANR values of the vehicles in the platoon and the error covariances at the previous discrete time ℓ−1. The trace of the updated covariance matrix for the secondary vehicle is expected to be much larger than that of the primary vehicle. This is attributed to the fact that the SANR of the sounding sample for the secondary vehicle is smaller than that of the primary vehicle. Consequently, this difference in the updated covariance matrices will continue to grow due to the unsuppressed errors that accumulate during the state transition processes. The estimation errors of the secondary vehicle could significantly impact the average vehicle tracking performance.

To address the expected lower estimation performance for the secondary vehicle, we propose a vehicle tracking algorithm that leverages the shared driving plans among platoon vehicles. In this section, we consider a platoon consisting of two vehicles. Although we focus on a scenario with two vehicles, the proposed algorithm can be readily extended to scenarios with more than two vehicles in the platoon. The first objective is to develop a strategy that exploits the state estimation results for the primary vehicle during the state estimation process for the secondary vehicles. As demonstrated in (20) and (21), the error covariance term, ${\hat{Q}}_{1}^{\ell |\ell -1}$, is common to both the primary and secondary vehicles. In Section 4.1, we propose a method to use the updated covariance matrix of the primary vehicle in (20) when the RSU corrects the error terms in the predicted covariance matrix for the secondary vehicle in (21). The second objective is to adaptively control the quality of the received sounding samples by considering the accumulated error covariances of the vehicles. In Section 4.2, we propose an array partitioning algorithm that efficiently divides the array into sub-arrays for each vehicle, aiming to obtain sounding samples suitable for enhancing the average tracking performance.

### 4.1. Proposed Vehicle Tracking Algorithm for Vehicle Platoon System

First, the RSU focuses on correcting the common error, $b^{\ell }$, by using the high SANR sounding sample transmitted from the primary vehicle. As shown in Figure 2, the lower sub-UPA with dimensions $M\times N_{1}$ is utilized to obtain sounding samples to track the primary vehicle, while the upper sub-UPA with dimensions $M\times N_{2}$ is used to obtain sounding samples to track the secondary vehicle. In this sub-section, we assume that $N_{1}=\frac{N}{2}$ and $N_{2}=\frac{N}{2}$ RF chains are used to obtain sounding samples for the primary vehicle and the secondary vehicles, among $N=N_{1}+N_{2}$ RF chains in the hybrid beamforming architecture of Figure 2. The state vector and the covariance matrix of the primary vehicle, $\left({\hat{t}}_{1}^{\ell },{\hat{Q}}_{1}^{\ell }\right)$, are estimated based on the vehicle tracking algorithm in Section 3.1. Notice that the channel vector of the secondary vehicle in (8) is predicted by using the temporary state vector of the secondary vehicle, $A{\hat{t}}_{2}^{\ell -1}$. The state vector of the primary vehicle is then modeled by using $\left({\hat{t}}_{1}^{\ell },{\hat{Q}}_{1}^{\ell }\right)$, such that

(23) $$\begin{array}{c} t_{1}^{\ell }={\hat{t}}_{1}^{\ell }+e_{1}^{\ell }\;\;\;\mathrm{with}\;\;\;e_{1}^{\ell }∼N\left(0,{\hat{Q}}_{1}^{\ell }\right). \end{array}$$

Second, the RSU estimates the state vector of the secondary vehicle in (19), expressed in terms of the state vector of the primary vehicle and the state difference vector. The state vector of the primary vehicle has already been estimated in (23) by correcting the common error with a high SANR sounding sample. Therefore, the RSU can concentrate on estimating the state difference vector by utilizing the low SANR sounding sample from the secondary vehicle. The correction of the common error can be applied to the state prediction process of the secondary vehicle by substituting the updated state vector of the primary vehicle, ${\hat{t}}_{1}^{\ell }$, into (19). Then, the state vector and the covariance matrix of the secondary vehicle are predicted as

(24) $$\begin{array}{c} {\hat{t}}_{2}^{\ell |\ell -1}={\hat{t}}_{1}^{\ell }+{\hat{t}}_{d}^{\ell |\ell -1}\;\;\;\;\;\mathrm{and}\;\;\;\;\;{\hat{Q}}_{2}^{\ell |\ell -1}={\hat{Q}}_{1}^{\ell }+{\hat{Q}}_{d}^{\ell |\ell -1}, \end{array}$$

where ${\hat{t}}_{d}^{\ell |\ell -1}$ and ${\hat{Q}}_{d}^{\ell |\ell -1}$ are defined in (22). Continuing with the Kalman filtering process, the state vector and the covariance matrix of the secondary vehicle are updated as in [36,37],

(25) $$\begin{array}{cc} {\hat{t}}_{2}^{\ell } & ={\hat{t}}_{2}^{\ell |\ell -1}+{\tilde{K}}_{2}\left({\tilde{r}}_{2}-{\tilde{Z}}_{2}\left(\sqrt{\rho_{1}}{\hat{h}}_{1}^{\ell }+\sqrt{\rho_{2}}{\hat{h}}_{2}^{\ell |\ell -1}\right)\right), \end{array}$$

(26) $$\begin{array}{cc} {\hat{Q}}_{2}^{\ell } & =\left(I_{2}-\sqrt{\rho_{2}}{\tilde{K}}_{2}{\tilde{Z}}_{2}{\tilde{D}}_{2}\right)\left({\hat{Q}}_{1}^{\ell }+{\hat{Q}}_{d}^{\ell |\ell -1}\right), \end{array}$$

where ${\hat{h}}_{1}^{\ell }$ is predicted by using ${\hat{t}}_{1}^{\ell }$ in (23) and ${\hat{h}}_{2}^{\ell |\ell -1}$ is predicted by using ${\hat{t}}_{2}^{\ell |\ell -1}$ in (24).

Before computing the Kalman gain matrix, we take a closer look at the predicted covariance matrices in (21) and (26). The main difference between the covariance matrices is that ${\hat{Q}}_{1}^{\ell -1}$ is replaced with ${\hat{Q}}_{1}^{\ell }$ in the predicted covariance matrix ${\hat{Q}}_{2}^{\ell |\ell -1}$. Notice that $\mathrm{Tr}\left\{{\hat{Q}}_{1}^{\ell }\right\}\ll \mathrm{Tr}\left\{{\hat{Q}}_{1}^{\ell -1}\right\}$ because ${\hat{Q}}_{1}^{\ell }$ represents the residual errors that remain after correcting the errors in ${\hat{Q}}_{1}^{\ell -1}$ through the state update process of the primary vehicle. In the proposed algorithm, the RSU can focus on mitigating the additional errors in ${\hat{Q}}_{d}^{\ell |\ell -1}$, instead of correcting the common errors in ${\hat{Q}}_{1}^{\ell |\ell -1}$. For this reason, it is expected that the state prediction performance of the proposed algorithm would surpass that of the conventional tracking algorithm in (7). This improvement is attributed to the correction of the common error during the state update process of the primary vehicle.

We now compute the Kalman gain matrix in (25) and (26). The objective of the state update process is to correct the additional error, $c^{\ell }$, by designing a Kalman matrix that minimizes the error between the real state vector in (19) and the updated state vector in (25):

(27) $$\begin{array}{cc} e_{2}^{\ell } & =t_{2}^{\ell }-{\hat{t}}_{2}^{\ell }=\left(t_{1}^{\ell }-{\hat{t}}_{1}^{\ell }\right)+\left(t_{d}^{\ell }-{\hat{t}}_{d}^{\ell |\ell -1}\right)-{\tilde{K}}_{2}\left({\tilde{r}}_{2}-{\tilde{Z}}_{2}\left(\sqrt{\rho_{1}}{\hat{h}}_{1}^{\ell }+\sqrt{\rho_{2}}{\hat{h}}_{2}^{\ell |\ell -1}\right)\right)\\ \; & \overset{\left(a\right)}{≃}\left(I-\sqrt{\rho_{2}}{\tilde{K}}_{2}{\tilde{Z}}_{2}{\tilde{D}}_{2}\right)\left(e_{1}^{\ell }+e_{d}^{\ell |\ell -1}\right)-{\tilde{K}}_{2}{\tilde{Z}}_{2}\left(\sqrt{\rho_{1}}{\tilde{D}}_{1}e_{1}^{\ell }+\frac{\sum_{v\in U}{\sqrt{\rho }}_{v}{\tilde{d}}_{v}^{\mathrm{nlos}}}{\sqrt{1+K}}\right)+{\tilde{K}}_{2}{\tilde{n}}_{2}, \end{array}$$

where ${\tilde{d}}_{v}^{\mathrm{nlos}}\in R^{2T}$ is the NLoS path of the *v*-th vehicle. In (27), (a) is derived by using $e_{1}^{\ell }=t_{1}^{\ell }-{\hat{t}}_{1}^{\ell }$, $e_{d}^{\ell |\ell -1}=t_{d}^{\ell }-{\hat{t}}_{d}^{\ell |\ell -1}$, and the linearized received sample that is approximated by

$$\begin{array}{c} {\tilde{r}}_{2}≃{\tilde{Z}}_{2}\left(\sqrt{\rho_{1}}\left({\hat{h}}_{1}^{\ell }+{\tilde{D}}_{1}e_{1}^{\ell }+\frac{{\tilde{d}}_{1}^{\mathrm{nlos}}}{\sqrt{1+K}}\right)+\sqrt{\rho_{2}}\left({\hat{h}}_{2}^{\ell |\ell -1}+{\tilde{D}}_{2}\left(e_{1}^{\ell }+e_{d}^{\ell |\ell -1}\right)+\frac{{\tilde{d}}_{2}^{\mathrm{nlos}}}{\sqrt{1+K}}\right)\right)+{\tilde{n}}_{2}. \end{array}$$

We define the error covariance matrix by using the error vector in (27), such that

(28) $$\begin{array}{cc} {\hat{Q}}_{2}^{\ell }=E\left[e_{2}^{\ell }{\left(e_{2}^{\ell }\right)}^{T}\right] & =\left(I-\sqrt{\rho_{2}}{\tilde{K}}_{2}{\tilde{Z}}_{2}{\tilde{D}}_{2}\right)\left({\hat{Q}}_{1}^{\ell }+{\hat{Q}}_{d}^{\ell |\ell -1}\right){\left(I-\sqrt{\rho_{2}}{\tilde{K}}_{2}{\tilde{Z}}_{2}{\tilde{D}}_{2}\right)}^{T}\\ \; & \;\;\;\;+{\tilde{K}}_{2}\left(\rho_{1}{\tilde{Z}}_{2}{\tilde{D}}_{1}{\hat{Q}}_{1}^{\ell }{\tilde{D}}_{1}^{T}{\tilde{Z}}_{2}^{T}+\left(\frac{\sum_{v\in U}\rho_{v}}{K+1}+1\right)\frac{I_{2}}{2}\right){\tilde{K}}_{2}^{T}, \end{array}$$

because $E\left[{\tilde{d}}_{v}^{\mathrm{nlos}}{\left({\tilde{d}}_{v}^{\mathrm{nlos}}\right)}^{T}\right]=\frac{I_{2MN_{2}}}{2}$, $E\left[{\tilde{Z}}_{2}{\tilde{d}}_{v}^{\mathrm{nlos}}{\left({\tilde{d}}_{v}^{\mathrm{nlos}}\right)}^{T}{\tilde{Z}}_{2}^{T}\right]=\frac{I_{2}}{2}$, and $E\left[{\tilde{n}}_{2}{\tilde{n}}_{2}^{T}\right]=\frac{I_{2}}{2}$. Similar to [36,37], the Kalman gain matrix must be designed to minimize the trace of the error covariance matrix in (28). The derivative of the trace of the covariance matrix is computed as

$$\begin{array}{cc} \frac{\partial \mathrm{Tr}\left\{{\hat{Q}}_{2}^{\ell }\right\}}{\partial {\tilde{K}}_{2}}= & -2\sqrt{\rho_{2}}\left({\hat{Q}}_{1}^{\ell }+{\hat{Q}}_{d}^{\ell |\ell -1}\right){\tilde{D}}_{2}^{T}{\tilde{Z}}_{2}^{T}+2{\tilde{K}}_{2}{\tilde{Z}}_{2}\left(\rho_{1}{\tilde{D}}_{1}{\hat{Q}}_{1}^{\ell }{\tilde{D}}_{1}^{T}\rho_{2}{\tilde{D}}_{2}\left({\hat{Q}}_{1}^{\ell }+{\hat{Q}}_{d}^{\ell |\ell -1}\right){\tilde{D}}_{2}^{T}\right){\tilde{Z}}_{2}^{T}\\ \; & +2{\tilde{K}}_{2}\left(\frac{\sum_{v\in U}\rho_{v}}{K+1}+1\right)\frac{I_{2}}{2}=0. \end{array}$$

The Kalman matrix is computed to solve the above equation:

$$\begin{array}{cc} {\tilde{K}}_{2}= & \sqrt{\rho_{2}}\left({\hat{Q}}_{1}^{\ell }+{\hat{Q}}_{d}^{\ell |\ell -1}\right){\tilde{D}}_{2}^{T}{\tilde{Z}}_{2}^{T}({\tilde{Z}}_{2}(\rho_{1}{\tilde{D}}_{1}{\hat{Q}}_{1}^{\ell }{\tilde{D}}_{1}^{T}\\ \; & +\rho_{2}{\tilde{D}}_{2}\left({\hat{Q}}_{1}^{\ell }+{\hat{Q}}_{d}^{\ell |\ell -1}\right){\tilde{D}}_{2}^{T}){\tilde{Z}}_{2}^{T}+\left(\frac{\sum_{v\in U}\rho_{v}}{K+1}+1\right)\frac{I_{2}}{2}{)}^{-1}. \end{array}$$

Lastly, the state difference vector and the covariance matrix are updated by using the updated information of the primary and secondary vehicles, such that

$$\begin{array}{c} {\hat{t}}_{d}^{\ell }={\hat{t}}_{2}^{\ell }-{\hat{t}}_{1}^{\ell }\;\;\;\;\;\mathrm{and}\;\;\;\;\;{\hat{Q}}_{d}^{\ell }={\hat{Q}}_{2}^{\ell }-{\hat{Q}}_{1}^{\ell }. \end{array}$$

### 4.2. Antenna Array Partitioning Algorithm

The vehicle tracking performance improves with the increase in the size of the channels for uplink sounding because the RSU can leverage the enhanced spatial multiplexing gains with more antennas. Assuming that the number of antenna elements in the horizontal domain is fixed as $\overset{ˇ}{M}$, the SANR in Proposition 1 is monotonically increasing over the number of RF chains because its first derivative is always positive, such as $\frac{\partial \gamma (\overset{ˇ}{M},N)}{\partial N}=\kappa \left(4N-3\right)\geq 0$, because N>0 and κ≥0. In the proposed vehicle tracking system, vehicles in a platoon share a limited number of RF chains used to obtain sounding samples because they utilize a common frequency band for uplink sounding. Since the number of RF chains is limited as $N=N_{1}+N_{2}$, as depicted in Figure 2, if the RSU utilizes the increased sub-array to enhance the tracking performance of the primary vehicle, the tracking performance of the secondary vehicle deteriorates, and vice versa. The optimal scenario for RF chain allocation, denoted as $\left({\overset{ˇ}{N}}_{1},{\overset{ˇ}{N}}_{2}\right)$, should be defined to maximize the expected average vehicle tracking performance at the next discrete time. To minimize the average error probability (maximize the average tracking performance), the quality of the received sounding samples should be adaptively controlled by considering the accumulated error covariances of the vehicles in the platoon, $\left({\hat{Q}}_{1}^{\ell -1},{\hat{Q}}_{2}^{\ell -1}\right)$. In the proposed beamforming architecture, we can control the SANRs by allocating the RF chains in Figure 2 to the vehicles in the driving platoon.

In this paper, we predict the average vehicle tracking performance based on the sum of trace of the updated error covariance matrices. In the proposed algorithm, the optimal scenario for RF chain allocation is defined by solving the following optimization problem. (Similar to the training model in [41], an array partitioning algorithm can be implemented based on deep Q-networks to alleviate the computational burden associated with solving optimization problems in rapidly changing environments.)

(29) $$\begin{array}{cc} {\overset{ˇ}{N}}_{1} & =\underset{\overset{˘}{N}\in \left\{1,\ldots ,N-1\right\}}{arg min}\mathrm{Tr}\left\{{\left({\hat{Q}}_{1}^{\ell }\right)}^{\overset{˘}{N}}\right\}+\mathrm{Tr}\left\{{\left({\hat{Q}}_{2}^{\ell }\right)}^{N-\overset{˘}{N}}\right\}\\ \; & \overset{\left(a\right)}{=}\underset{\overset{˘}{N}\in \left\{1,\ldots ,N-1\right\}}{arg max}\mathrm{Tr}\left\{\sqrt{\rho_{1}}{\tilde{K}}_{1}^{\overset{˘}{N}}{\tilde{Z}}_{1}^{\overset{˘}{N}}{\tilde{D}}_{1}^{\overset{˘}{N}}{\hat{Q}}_{1}^{\ell |\ell -1}\right\}+\mathrm{Tr}\left\{\sqrt{\rho_{2}}{\tilde{K}}_{2}^{N-\overset{˘}{N}}{\tilde{Z}}_{2}^{N-\overset{˘}{N}}{\tilde{D}}_{2}^{N-\overset{˘}{N}}{\hat{Q}}_{2}^{\ell |\ell -1}\right\}\\ \; & \overset{\left(b\right)}{≃}\underset{\overset{˘}{N}\in \left\{1,\ldots ,N-1\right\}}{arg max}e\left\{\rho_{1}{\left(\Lambda^{\overset{˘}{N}}\right)}^{-1}D_{1}^{\overset{˘}{N}}\left(:,1\right){\left({\hat{Q}}_{1}^{\ell |\ell -1}\left(1,1\right)\right)}^{2}D_{1}^{\overset{˘}{N}}{\left(:,1\right)}^{H}\right\}\\ \; & \;\;\;\;\;\;\;\;\;\;\;\;\;\;\;\;\;\;\;\;\;\;\;\;\;\;\;\;\;\;+e\left\{\rho_{2}{\left(\Lambda^{N-\overset{˘}{N}}\right)}^{-1}D_{2}^{N-\overset{˘}{N}}\left(:,1\right){\left({\hat{Q}}_{2}^{\ell |\ell -1}\left(1,1\right)\right)}^{2}D_{2}^{N-\overset{˘}{N}}{\left(:,1\right)}^{H}\right\}, \end{array}$$

where $\Lambda^{L}=\sum_{v\in U}\rho_{v}D_{v}^{L}\left(:,1\right){\hat{Q}}_{v}^{\ell |\ell -1}\left(1,1\right)D_{v}^{L}{\left(:,1\right)}^{H}+\left(\frac{\sum_{v\in U}\rho_{v}}{K+1}+1\right)I_{ML}$, and ${\overset{ˇ}{N}}_{2}=N-{\overset{ˇ}{N}}_{1}$. Assuming that *L* RF chains are allocated to the *v*-th vehicle, the updated covariance matrix, combiner, and Jacobian matrix are, respectively, denoted by ${\left({\hat{Q}}_{v}^{\ell }\right)}^{L}$, ${\tilde{Z}}_{v}^{L}$, ${\tilde{D}}_{v}^{L}$. In (29), (a) is derived because

$$\begin{array}{c} \underset{1\leq \overset{˘}{N}<N}{arg min}\left(I_{2}-\sqrt{\rho_{u}}{\tilde{K}}_{u}^{\overset{˘}{N}}{\tilde{Z}}_{u}^{\overset{˘}{N}}{\tilde{D}}_{u}^{\overset{˘}{N}}\right){\hat{Q}}_{u}^{\ell |\ell -1}=\underset{1\leq \overset{˘}{N}<N}{arg max}\sqrt{\rho_{u}}{\tilde{K}}_{u}^{\overset{˘}{N}}{\tilde{Z}}_{u}^{\overset{˘}{N}}{\tilde{D}}_{u}^{\overset{˘}{N}}{\hat{Q}}_{u}^{\ell |\ell -1}, \end{array}$$

and (b) is derived by reformulating the object function in the complex domain, as in (14). The object function in (b) is written by plugging the complex combiners and the Kalman matrices into (29).

It is necessary to obtain the updated covariance matrix of the primary vehicle, ${\hat{Q}}_{1}^{\ell }$, in order to realize the predicted covariance matrix for the secondary vehicle ${\hat{Q}}_{2}^{\ell |\ell -1}={\hat{Q}}_{1}^{\ell }+{\hat{Q}}_{d}^{\ell |\ell -1}$. However, the covariance of the primary vehicle cannot be updated at the time of allocating the RF chains in (29). In the state update process of the secondary vehicle, the RSU should focus on mitigating the individual error transition vector. For these reasons, in the proposed algorithm, the predicted covariance in (29) is rewritten as ${\hat{Q}}_{2}^{\ell |\ell -1}={\hat{Q}}_{d}^{\ell |\ell -1}$, without considering the updated covariance matrix of the primary vehicle.

Lastly, the RSU exploits the selected set of RF chains, $\left({\overset{ˇ}{N}}_{1},{\overset{ˇ}{N}}_{2}\right)=\left({\overset{ˇ}{N}}_{1},N-{\overset{ˇ}{N}}_{1}\right)$, to obtain sounding samples from the vehicles in the platoon. We assume that the RSU reconstructs the set of RF chains for $\Omega T_{s}$ seconds. The proposed tracking methods in Section 4 are summarized in Algorithm 1.
**Algorithm 1** Vehicle tracking algorithm for driving platoon.**I. Initialization**1: Initial state vector, ${\hat{t}}_{u}^{0}=t_{u}^{0}+e_{u}^{\epsilon }$ with $t_{u}^{0}={\left[x_{u}^{0},v_{u}^{0}\right]}^{T},\;u\in \left\{1,2\right\}$2: Initial difference vector, ${\hat{t}}_{d}^{0}={\hat{t}}_{2}^{0}-{\hat{t}}_{1}^{0}={\left[\Delta_{x}^{0},\Delta_{y}\right]}^{T}+\left(e_{2}^{\epsilon }-e_{1}^{\epsilon }\right)$3: Initial covariance matrix, ${\hat{Q}}_{u}^{0}={\left(\sigma_{\epsilon }T_{s}\right)}^{2}t_{u}^{0}{\left(t_{u}^{0}\right)}^{T}$**II. RF chain allocation, $\left(N_{1},N_{2}\right)$, in Section 4.2****III. State prediction and estimation for primary vehicle using $N_{1}$ RF chains**4: Predict state vector and covariance, ${\hat{t}}_{1}^{\ell |\ell -1}=A{\hat{t}}_{1}^{\ell -1}\;\;\;\&\;\;\;\;{\hat{Q}}_{1}^{\ell |\ell -1}=A{\hat{Q}}_{1}^{\ell -1}A^{T}+Q_{b}$5: Update state vector, ${\hat{t}}_{1}^{\ell }={\hat{t}}_{1}^{\ell |\ell -1}+{\tilde{K}}_{1}\left({\tilde{r}}_{1}-{\tilde{Z}}_{1}\left(\sqrt{\rho_{1}}{\hat{h}}_{1}^{\ell |\ell -1}+\sqrt{\rho_{2}}{\hat{h}}_{2}^{\ell |\ell -1}\right)\right)$6: Update covariance matrix, ${\hat{Q}}_{1}^{\ell }=\left(I_{2}-\sqrt{\rho_{1}}{\tilde{K}}_{1}{\tilde{Z}}_{1}{\tilde{D}}_{1}\right){\hat{Q}}_{1}^{\ell |\ell -1}$**IV. State prediction and estimation for secondary vehicle using $N_{2}$ RF chains**7: Predict difference vector and covariance, ${\hat{t}}_{d}^{\ell |\ell -1}=A{\hat{t}}_{d}^{\ell -1}\;\;\;\&\;\;\;\;{\hat{Q}}_{d}^{\ell |\ell -1}=A{\hat{Q}}_{d}^{\ell -1}A^{T}+Q_{c}$8: Predict state vector and covariance, ${\hat{t}}_{2}^{\ell |\ell -1}={\hat{t}}_{1}^{\ell }+{\hat{t}}_{d}^{\ell |\ell -1}\;\;\;\&\;\;\;\;{\hat{Q}}_{2}^{\ell |\ell -1}={\hat{Q}}_{1}^{\ell }+{\hat{Q}}_{d}^{\ell |\ell -1}$9: Update state vector, ${\hat{t}}_{2}^{\ell }={\hat{t}}_{2}^{\ell |\ell -1}+{\tilde{K}}_{2}\left({\tilde{r}}_{2}-{\tilde{Z}}_{2}\left(\sqrt{\rho_{1}}{\hat{h}}_{1}^{\ell }+\sqrt{\rho_{2}}{\hat{h}}_{2}^{\ell |\ell -1}\right)\right)$10: Update covariance matrix, ${\hat{Q}}_{2}^{\ell }=\left(I_{2}-\sqrt{\rho_{2}}{\tilde{K}}_{2}{\tilde{Z}}_{2}{\tilde{D}}_{2}\right)\left({\hat{Q}}_{1}^{\ell }+{\hat{Q}}_{d}^{\ell |\ell -1}\right)$**V. State difference estimation**11: Update state difference vector, ${\hat{t}}_{d}^{\ell }={\hat{t}}_{2}^{\ell }-{\hat{t}}_{1}^{\ell }$12: Update covariance matrix, ${\hat{Q}}_{d}^{\ell }={\hat{Q}}_{2}^{\ell }-{\hat{Q}}_{1}^{\ell }$

## 5. Simulation Results

This section presents simulation results to validate the proposed vehicle tracking and array partitioning algorithms. We first present the system and channel parameters considered in the channel sounding system of (1). For the uplink channel sounding, each vehicle transmits radio signals at a center frequency of 27 GHz, utilizing a bandwidth of 20 MHz. The transmit power of each vehicle, ϱ, is distributed over 20 MHz. The power of average noise over the 20 MHz bandwidth is computed as $\sigma_{n}^{2}=-174+10\log_{10}\left(20\times 10^{6}\right)≃-101$ dBm. The average SNR is then calculated by assuming that the path loss exponent parameter is set to two. We consider a UPA with *T* = 48, 96 antenna elements and N=6,8 RF chains, in which M=8,12 antenna elements in each row of the horizontal domain are connected to a single RF chain. The antenna element spacing parameter is ν=2 and the combiner switching parameter is Ω=4. For the numerical studies, we randomly generate 50,000 sets of millimeter wave (mmWave) channels consisting of an LoS path and an NLoS path with a Rician *K*-factor, K=11 dB.

We next present the parameters in the street geometry model, depicted in Figure 1. We consider a realistic street geometry with four-lane roads and RSU deployment, in which the position of the ℓ-th lane in the *y*-axis is 5+3.5ℓm and the height difference between the RSU and the tops of the vehicles is h=10m. The platoon consists of two vehicles driving with an initial velocity of $v_{0}=60\;\mathrm{km}/h$ (16.67 m/s). The primary vehicle is in the first lane and the secondary vehicle is in the third lane. The initial positions of the vehicles are set to $\left(x_{2}^{0},y_{2}\right)=\left(x_{1}^{0}+\Delta_{x}^{0},y_{1}+\Delta_{y}\right)$, with $\Delta_{x}^{0}=80\;m$, $\Delta_{y}=3.5\times 2\;m$, and $y_{1}=8.5\;m$. We consider two different initial positions of the primary vehicle on the *x*-axis, i.e., $x_{1}^{0}=50$ m and $x_{1}^{0}=100$ m. Note that $x_{u}^{0}$ and $y_{u}$ are the initial positions of the *u*-th vehicle on the *x*-axis and *y*-axis. We also present the parameters in the state transition model of (6). The sampling period is set to $T_{s}=10$ ms. Furthermore, the error transition parameters are set to $\left\{\sigma_{b},\sigma_{c}\right\}=\left\{10^{-0.5},10^{-1}\right\}$ and the feedback error parameter is set to $\sigma_{\epsilon }=10^{-1}$.

**Figure 3:**
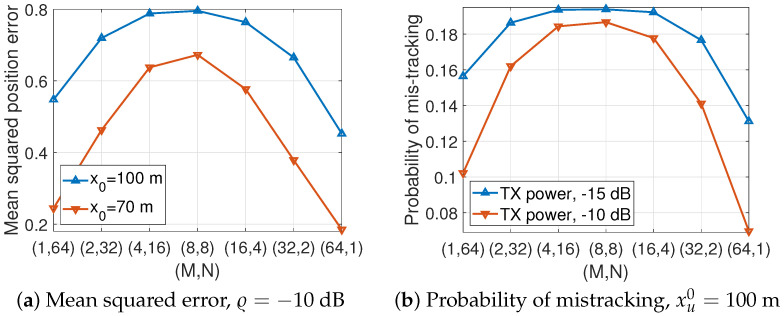
Tracking performance against antenna array layout.

We next validate the proposed array configuration framework presented in Section 3.2. The vehicle tracking performance is averaged through 50,000 Monte Carlo simulations. We consider the four-lane road so that ${\bar{y}}^{2}=\frac{\sum_{\ell =1}^{4}{\left(5+3.5\ell \right)}^{2}}{4}\;m^{2}$, which is greater than $h^{2}=10^{2}\;m^{2}$. The vehicle tracking performance is evaluated by using the mean squared position error, $\Upsilon_{x}=E[|x_{u}^{\ell }-{\hat{x}}_{u}^{\ell }{|}^{2}]$, and the probability of mistracking, $\Psi_{x}=\mathrm{Pr}(|x_{u}^{\ell }-{\hat{x}}_{u}^{\ell }{|}^{2}<1\;m^{2})$, with u∈U≐{1,2}. In Figure 3, the system using the horizontal ULA shows the best vehicle tracking performance, as expected in Section 3.2. It is also verified that the horizontal UPA with more columns than rows outperforms the vertical UPA with more rows than columns.

**Figure 4:**
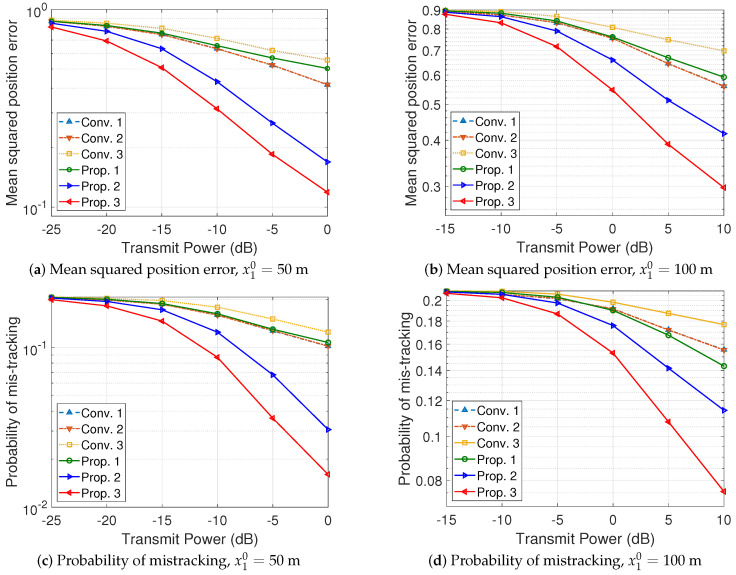
Performance evaluation of vehicle tracking systems, (M,N)=(8,6).

**Figure 5:**
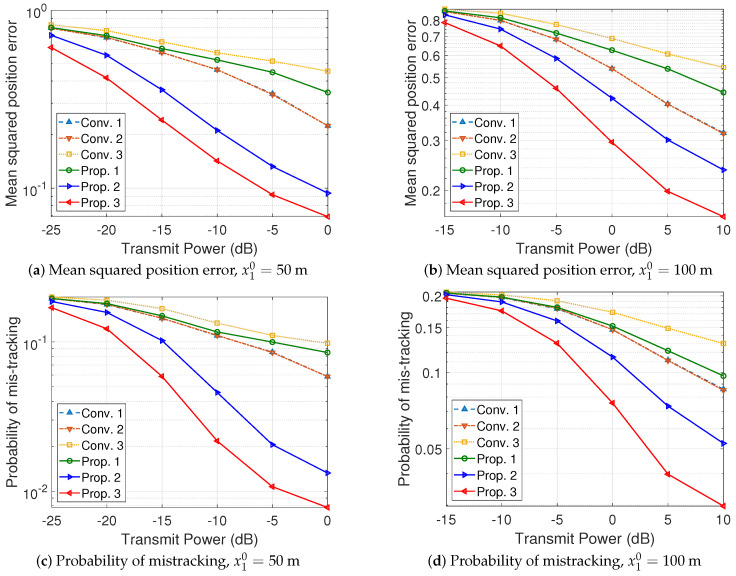
Performance evaluation of vehicle tracking systems, (M,N)=(12,8).

The position estimation performance of the vehicle tracking systems is evaluated in Figure 4 and Figure 5. The details of the vehicle tracking systems are summarized in Table 1. It must be noted that the *Conv. 1* and *Conv. 2* systems exploit an M×N size antenna array for the sounding of each vehicle, while the *Conv. 3* and *Prop. 1* systems exploit an $M\times \frac{N}{2}$ size antenna array for the sounding of each vehicle, and the *Prop. 2* and *Prop. 3* systems exploit an $M\times N_{u}$ size antenna array for the sounding of the *u*-th vehicle. The *Conv. 1* and *Conv. 2* systems utilize two frequency slots, while the other systems utilize a single frequency slot. Because the *Conv. 1* and *Conv. 2* systems allocate each frequency slot to track a vehicle, *N* sounding samples, obtained by using all M×1 size horizontal ULAs in Figure 2, can be used to track a single vehicle. Assuming $N_{1}=N_{2}=\frac{N}{2}$, the *Conv. 3* system, exploiting an M×1 horizontal ULA, uses $\frac{N}{2}$ sounding samples to track the trajectories of a vehicle in the platoon. Furthermore, the *Prop. 1* and *Prop. 2* systems exploit the $M\times \frac{N}{2}$ sub-UPA to update the state vector of a vehicle in the platoon (a set of $\frac{N}{2}$ RF chains is used to obtain a single sounding sample). The *Prop. 3* system is designed based on the vehicle tracking algorithm in Section 4.1 and the array partitioning algorithm in Section 4.2. Assuming $N=N_{1}+N_{2}$, the *Prop. 3* system adjusts the sizes of the sub-UPAs, $\left(N_{1},N_{2}\right)$, to obtain sounding samples for vehicles in the platoon based on the estimated covariance matrices.

As depicted in Figure 4 and Figure 5, the tracking performance of the systems utilizing the UKF and EKF is negligible. The *Prop. 1* system using the UPA outperforms the *Conv. 3* system using the ULA with multiple samples because it can fully exploit the benefits of spatial multiplexing gains and utilize angular variations in both the horizontal and vertical domains. Furthermore, the *Prop. 2* system enhances the estimation performance of the secondary vehicle because the common error has been corrected in the state update process of the primary vehicle. The proposed tracking algorithm enhances the position estimation performance for secondary vehicles, thereby contributing to an overall improvement in the average estimation performance. Lastly, the proposed array partitioning algorithm in the *Prop. 3* system further improves the average estimation performance by adjusting the sizes of the sub-UPAs according to the SANRs and the accumulated transition errors of the vehicles in the platoon. For example, in the case of (M,N)=(12,8), ϱ=0 dB, and $x_{1}^{0}=100$ m as shown in Figure 5d, the probability of beam mistracking in the *Conv. 3*, *Prop. 1*, *Prop. 2*, and *Prop. 3* systems is given by 0.172, 0.152, 0.115, and 0.0758, respectively. It is confirmed that the *Prop. 2* system, designed for platooned traffic scenarios, enhances the tracking performance by 6.88% and 4.36% compared to the *Conv. 1* and *Prop. 1* systems, respectively. From the perspective of the probability of correct tracking, the performance enhancements are calculated as $\frac{\left(1-0.115)-(1-0.172\right)}{\left(1-0.172\right)}\times 100\%$ and $\frac{\left(1-0.115)-(1-0.152\right)}{\left(1-0.152\right)}\times 100\%$, respectively. It is also verified that the proposed antenna array partitioning algorithm in the *Prop. 3* system further enhances the tracking performance by an additional 4.43% compared to the *Prop. 2* system, which does not incorporate the antenna array partitioning algorithm.

## 6. Conclusions

We developed EKF-based vehicle tracking algorithms by considering the 2D UPA layout and the vehicle platoon scenario. First, we established an analytical framework to guide the optimal configuration of the UPA for effective vehicle tracking within V2I networks. Following the proposed analytical guideline, we confirmed that the most suitable UPA layout to maximize the vehicle tracking performance in a variety of real-world road environments is a horizontally elongated rectangular UPA, with a length along the horizontal axis greater than that along the vertical axis. Second, we developed a method for the effective tracking of vehicle trajectories by leveraging the similarity of the paths among the vehicles forming the driving platoon. The EKF-based vehicle tracking system was redesigned to harness the advantages of the UPA by considering the specialized state transition model for vehicle platoon scenarios. The proposed vehicle tracking system, customized for platoon driving scenarios, demonstrated improved position estimation performance compared to conventional systems that independently estimate the trajectory of each vehicle. Lastly, an array partitioning algorithm was proposed to efficiently divide the array into sub-arrays for each platoon vehicle, with the goal of enhancing the average tracking performance. Numerical studies confirmed that the proposed vehicle tracking for platoon driving scenarios enhances the accuracy of the tracking system by leveraging shared driving plans. Furthermore, it was verified that the proposed array partitioning algorithm improves the average tracking performance by judiciously allocating limited wireless resources, considering the accumulated estimation errors and channel conditions. While this paper focused solely on the UPA, future research could explore the development of an analytical framework for the design of 2D array layouts beyond the rectangular UPA. Moreover, it would be an interesting future research topic to develop physical layer techniques that can enhance the security of the state estimation process in V2I networks, without relying on cryptography.

## Figures and Tables

**Table 1:** Vehicle tracking systems for numerical results in Figure 4 and Figure 5.

Tracking System	Reference	Array Layout	Number of Freq. Slots for Channel Sounding	Array Size	Number of Sounding Samples
*Conv. 1*	UKF in [18]	ULA	2	M×1	N
*Conv. 2*	EKF in [15]	ULA	2	M×1	N
*Conv. 3*	EKF in [16]	ULA	1	M×1	$\frac{N}{2}$
*Prop. 1*	EKF in Section 3.1	UPA	1	$M\times \frac{N}{2}$	1
*Prop. 2*	EKF in Section 4.1	UPA	1	$M\times N_{u}$	1
*Prop. 3*	EKF in Section 4.1 with array partitioning in Section 4.2	UPA	1	$M\times N_{u}$	1

## Data Availability

Data are contained within the article.

## Abbreviations

The following abbreviations are used in this manuscript:

5G	Fifth Generation
6G	Sixth Generation
V2X	Vehicle-to-Everything
V2I	Vehicle-to-Infrastructure
RSU	Roadside Unit
EKF	Extended Kalman Filter
UKF	Unscented Kalman Filter
mmWave	Millimeter Wave
RF	Radio Frequency
AoD	Angle-of-Departure
LoS	Line-of-Sight
NLoS	Non-Line-of-Sight
2D	Two-Dimensional
3D	Three-Dimensional
UPA	Uniform Planar Array
ULA	Uniform Linear Array
SNR	Signal-to-Noise Ratio
SANR	Signal-Plus-Angular-Derivative-to-Noise-Ratio
